# EpxMedTracking: Feasibility Evaluation of an SMS-Based Medication Adherence Tracking System in Community Practice

**DOI:** 10.2196/resprot.7223

**Published:** 2017-05-15

**Authors:** Christopher Tricarico, Robert Peters, Avik Som, Kavon Javaherian, Will Ross

**Affiliations:** ^1^ Washington University in St. Louis School of Medicine St. Louis, MO United States; ^2^ Epharmix Research Center St. Louis, MO United States

**Keywords:** medication adherence, eHealth, text messaging

## Abstract

**Background:**

Medication adherence remains a difficult problem to both assess and improve in patients. It is a multifactorial problem that goes beyond the commonly cited reason of forgetfulness. To date, eHealth (also known as mHealth and telehealth) interventions to improve medication adherence have largely been successful in improving adherence. However, interventions to date have used time- and cost-intensive strategies or focused solely on medication reminding, leaving much room for improvement in using a modality as flexible as eHealth.

**Objective:**

Our objective was to develop and implement a fully automated short message service (SMS)-based medication adherence system, EpxMedTracking, that reminds patients to take their medications, explores reasons for missed doses, and alerts providers to help address problems of medication adherence in real time.

**Methods:**

EpxMedTracking is a fully automated bidirectional SMS-based messaging system with provider involvement that was developed and implemented through Epharmix, Inc. Researchers analyzed 11 weeks of de-identified data from patients cared for by multiple provider groups in routine community practice for feasibility and functionality. Patients included were those in the care of a provider purchasing the EpxMedTracking tool from Epharmix and were enrolled from a clinic by their providers. The primary outcomes assessed were the rate of engagement with the system, reasons for missing doses, and self-reported medication adherence.

**Results:**

Of the 25 patients studied over the 11 weeks, 3 never responded and subsequently opted out or were deleted by their provider. No other patients opted out or were deleted during the study period. Across the 11 weeks of the study period, the overall weekly engagement rate was 85.9%. There were 109 total reported missed doses including “I forgot” at 33 events (30.3%), “I felt better” at 29 events (26.6%), “out of meds” at 20 events (18.4%), “I felt sick” at 19 events (17.4%), and “other” at 3 events (2.8%). We also noted an increase in self-reported medication adherence in patients using the EpxMedTracking system.

**Conclusions:**

EpxMedTracking is an effective tool for tracking self-reported medication adherence over time. It uniquely identifies actionable reasons for missing doses for subsequent provider intervention in real time based on patient feedback. Patients enrolled on EpxMedTracking also self-report higher rates of medication adherence over time while on the system.

## Introduction

### Overview

As medical innovation continues to give us powerful tools to treat and prevent disease, one major obstacle between patients and the benefits of treatment has been medication nonadherence. Nonadherence rates vary widely among different disease contexts and patient populations, but estimates of nonadherence typically range from 50% to 80% [1] with nonadherence notably elevated in asymptomatic [1] and chronic [2] conditions. This ultimately has a major impact on clinical outcomes as nonadherence is estimated to be a major factor in 125,000 deaths per year [3]. Medication nonadherence is also an important target for improvement in the context of increasing cost-containment pressures in the United States. For example, medication nonadherence has been estimated to cause approximately 10% of total hospital admissions and results in an estimated cost to the health care system of $100 to $289 billion annually [3].

Because by definition nonadherence is a problem that occurs outside of the clinic when patients are away from their providers, one strategy used to combat this problem in recent years has been eHealth. eHealth uses telecommunications and computer technology to enable provider-patient communication across geographic boundaries [4]. In particular, the growing ubiquity of telecommunication and mobile devices provides a novel route for provider-patient interaction at a distance and a powerful tool to reduce nonadherence. It is estimated that by 2017, 8 billion mobile phones with short message service (SMS) text messaging capability will be in use with especially high prevalence in certain medically vulnerable populations such as minorities and people in developing countries [5]. The use of basic SMS text messaging with such devices is an especially attractive option for eHealth intervention as it offers a familiar, nonintrusive, and easy-to-use tool for increasing medication adherence.

### Prior Work

The use of text messages and phone calls to improve medication adherence has been extensively studied over the past 15 years, and one recent meta-analysis found 11 articles using SMS- and phone call–based interventions showing a cumulative increase in medication adherence of 22% (risk ratio 1.22, 95% CI 1.09-1.36) [6]. Studies have also shown that SMS text-based medication reminders can result in significantly higher levels of medication adherence, fewer hospital admissions, and lower mortality rates [7,8].

One important concept that has emerged from this literature has been that technologies that facilitate 2-way communication with providers are significantly more successful than unidirectional interventions [9] or simple electronic reminder devices [10]. While it seems clear based on these studies that SMS messages and phone calls can improve medication adherence, there remains little evidence about how to best use this methodology. The vast promise of this methodology hinges on further work to better understand and refine eHealth techniques and establish evidence-based protocols.

### Theory

In terms of thinking about how an SMS-based system could be optimized to address medication nonadherence, it may be helpful to consider the problem in the context of the framework proposed in the systematic review by Yap et al [11] on the barriers to medication adherence in older adults, in which they organize the reasons for nonadherence that have been proven to date into 5 categories: patient factors, medication factors, physician factors, system-based factors, and other factors. Many of the factors they identified include situations and patient beliefs that could conceivably be identified and addressed by an SMS- or phone call–based system (see Textbox 1).

**Factors shown to affect medication adherence that could be identified or addressed by a short message service– or phone call–based eHealth intervention.**

Patient factors:

- Depression
- Poor memory
- Anxiety
- Sleep disturbances
- Poor physical function
- Nonadherence to follow-ups
- Problem drinking
- Beliefs about medication
- Lack of threatening view of illness
- Lack of perceived benefit of medications
- Knowledge of chronicity of illness
- Knowledge of consequences of illness
- Lack of knowledge about condition
- Lack of medication knowledge
- Misunderstanding of verbal instructions

Medication factors:

- Multiple medications
- Logistical barriers to medication filling
- Adverse drug reactions

Physician factors:

- Poor communication
- Lack of involvement of patients

System-based factors:

- Lack of follow-up
- Lack of medication review
- Lack of patient education

Other factors:

- At least one previous episode of nonadherence

Given that Yap et al [11] identified 80 different factors shown to influence nonadherence, it seems clear that this problem is complex and multifactorial. While forgetfulness is often cited as a reason for medication nonadherence, Saberi et al [10] found that interventions relying solely on reminding HIV patients to take their antiretroviral therapy were largely not effective, leading them to speculate that the attributed forgetfulness was a simple excuse for more complex underlying reasons like stigma, depression, drug abuse, and lack of social support. Across all patient contexts, Brown et al [1] have also concluded that although most physicians believe nonadherence is due to forgetfulness or lack of access, it is often a deliberate choice by the patient.

In the design of any technology, it is critical that special attention is paid to end-user reactions to the technology. In this case both patients and providers are end-users. One theory known as the technology acceptance model has been shown to predict a substantial portion of the actual use of information technology in the health care setting [12]. As such, an effective eHealth intervention should pay special attention to key variables in the technology acceptance model such as perceived usefulness and perceived ease of use.

Because the reasons for nonadherence among individual patients and situations are nuanced and context-driven, designing an appropriate eHealth intervention provides a significant challenge. A successful intervention must be able to deconstruct and reorganize the complex reasons for nonadherence in a way that is easily interpretable for providers. Furthermore, the use of bidirectional SMS or phone call messaging to improve medication adherence demands that patients and providers be willing to respond and engage with the system for long periods of time.

### Hypothesis

Recognizing the principles that have emerged from the literature, we set out to design a stand-alone automated intervention that would be more functional and cost effective than the current standard of care. The Epharmix Medication Tracking system, hereafter referred to as EpxMedTracking, is a medication adherence eHealth intervention that both reminds patients when they are supposed to take their medication and goes through a “differential diagnosis” of why that patient missed their dose and subsequently alerts their providers. In doing so, we hoped to give providers real-time insight into when a patient is feeling sick, out of meds, or may have mistaken beliefs about their medication regimen. By identifying patients who are not taking their medication because they feel sick or because the medication makes them feel sick, the system also has the capacity to detect adverse drug reactions as they occur. In this study, we aimed to evaluate the feasibility of the EpxMedTracking system as a tool to improve medication adherence in terms of its acceptability by patients and its ability to determine why patients are missing doses.

## Methods

### Study Design

#### Data Collection

To assess the feasibility of using EpxMedTracking we analyzed de-identified aggregate data provided by Epharmix, an outside telehealth vendor that specializes in designing condition-specific, automated text messages that are optimized for both clinical utility and patient engagement. We piloted the system for 17.5 weeks and then made content changes based on feedback from current users and feedback from a focus group of other low socioeconomic status (SES) patients (specifically patients with HIV) and their case managers. This feedback was used to inform the design of the smart engagement module which included specific message wording and rotating greeting messages to improve patient engagement with the system. We specifically chose to solicit input from HIV patients and their case managers because this is a patient population where medication adherence is critical. As such we see it as an important population for future implementation of EpxMedTracking and studies to validate its efficacy. Following the implementation of those changes the official study period began.

#### Participants

Because we used convenience sampling, we did not use specific eligibility criteria. Literacy was also not an eligibility criterion as all text message wording was scored at less than a 6th grade reading level as determined by the Flesch-Kincaid Grade Level scale. SMS literacy was a de facto eligibility criteria.

Patients included in both the pilot and study came from the commercial implementation of the system by several different provider groups that purchased the Epharmix service to improve the management of their patients. Thus we used convenience sampling of the data that we had access to, and recruitment to the study was handled by the providers themselves and not by the research team or Epharmix. Participants were therefore recruited remotely and offline from a clinic and were anonymous to the research team and Epharmix. Because this was a quality improvement project and not an institutional review board–approved trial, participants were not required to fill out informed consent and were briefed for recruitment at the discretion of their provider. Having multiple identities was not possible given the technical capacity of the Epharmix servers. Institutional affiliation with Washington University was not displayed to patients. Provider groups included in this study include two groups from St. Louis, Missouri, and one group from Georgia.

#### Intervention

The intervention was developed as a collaboration between independent researchers and Epharmix, an independent company and owner of the software. Epharmix has sponsored a research center at Washington University School of Medicine to foster the design and development of eHealth interventions. We piloted the system for a period of 17.5 weeks (123 days) and 3324 total sessions. During the pilot period, we solicited feedback from patients enrolled in the system and found that many of them reported that interacting with the system felt like they were talking to a machine. Based on this feedback we added a smart engagement module, which includes encouraging messaging and phrasing that enables the service to take on the role of patient advocate and supporter. The smart engagement module also included rotating greeting messages (see Multimedia Appendix 1) to initiate text message interactions to add a degree of novelty for patients. The smart engagement module was included in the EpxMedTracking intervention for all patients using the system following the pilot period. Particular attention was also paid to designing messages that would be effective even for patients with low health literacy. In designing the smart engagement module, we also interviewed 5 low SES HIV patients not currently enrolled in the system and their case managers to solicit input on message wording and what they would want out of an SMS medication tracking system. We chose this particular demographic because we felt it would be a high-yield demographic for future use of the system and future studies to verify its efficacy using clinical outcome measures, such as tracking viral titers. The categories of nonadherence included “I forgot,” “I felt sick,” “out of meds,” “I am no longer taking,” “I felt better,” and “other.” These categories of nonadherence were designed in collaboration with two practicing physicians.

After the smart engagement module was added, we ended the pilot period and began the study period immediately with no washout period. For the pilot study, 26 patients were enrolled. Of those, 9 were deleted by their provider including 2 that opted out on their own before being deleted. Of those patients, 8 had scheduled stops (whereby the provider only prescribed EpxMedTracking for a certain period of time after which they discontinued use of the system). A total of 9 patients from the pilot continued on to the study of EpxMedTracking, and 16 new patients were added after the pilot during the study period. Thus there were 25 total patients included in the study over a period of 11 weeks. The 9 patients studied in the official study period that had already been enrolled in the pilot were enrolled in the pilot for an average of 35.2 (SD 22.9) weeks.

**Figure 1:**
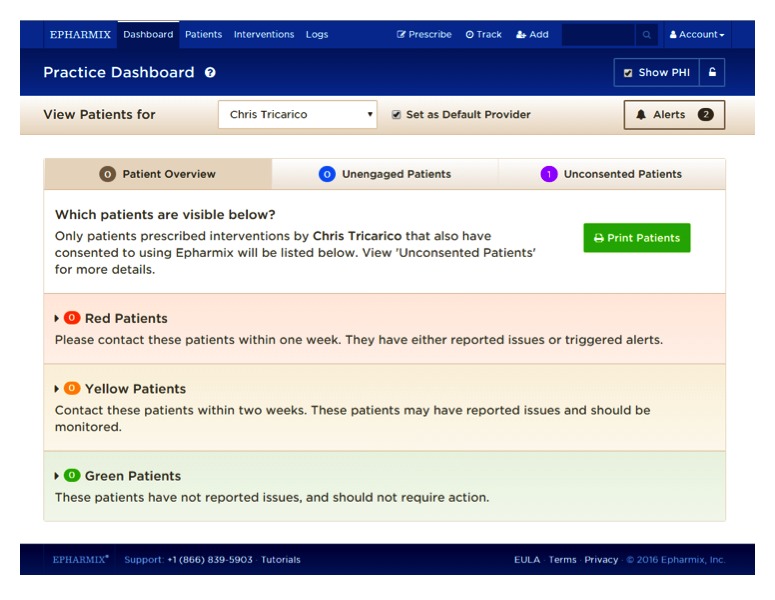
Web portal.

Patients accessed the app by receiving an SMS message on their phone. Messages were free of charge to all patients and patients did not need to be a part of a special group to receive them. Providers accessed the platform via the Web portal [13] directly. A screenshot from the Web portal is depicted in Figure 1.

Epharmix monitors the message gateway status, and any major technical errors are accounted for when calculating the responses. In the case of the study, no major errors occurred. A patient who does not receive messages due to technology-based errors (poor cell reception, lack of SMS service, incorrect phone number entered into the system) would be displayed on the provider dashboard as unengaged, prompting provider follow-up to solve the technical problem. Epharmix receives delivery status reports for all messages sent to ensure their deliverability, and future iterations of EpxMedTracking will generate an internal alert at Epharmix for multiple undelivered messages to address these technical problems without provider involvement.

**Figure 2:**
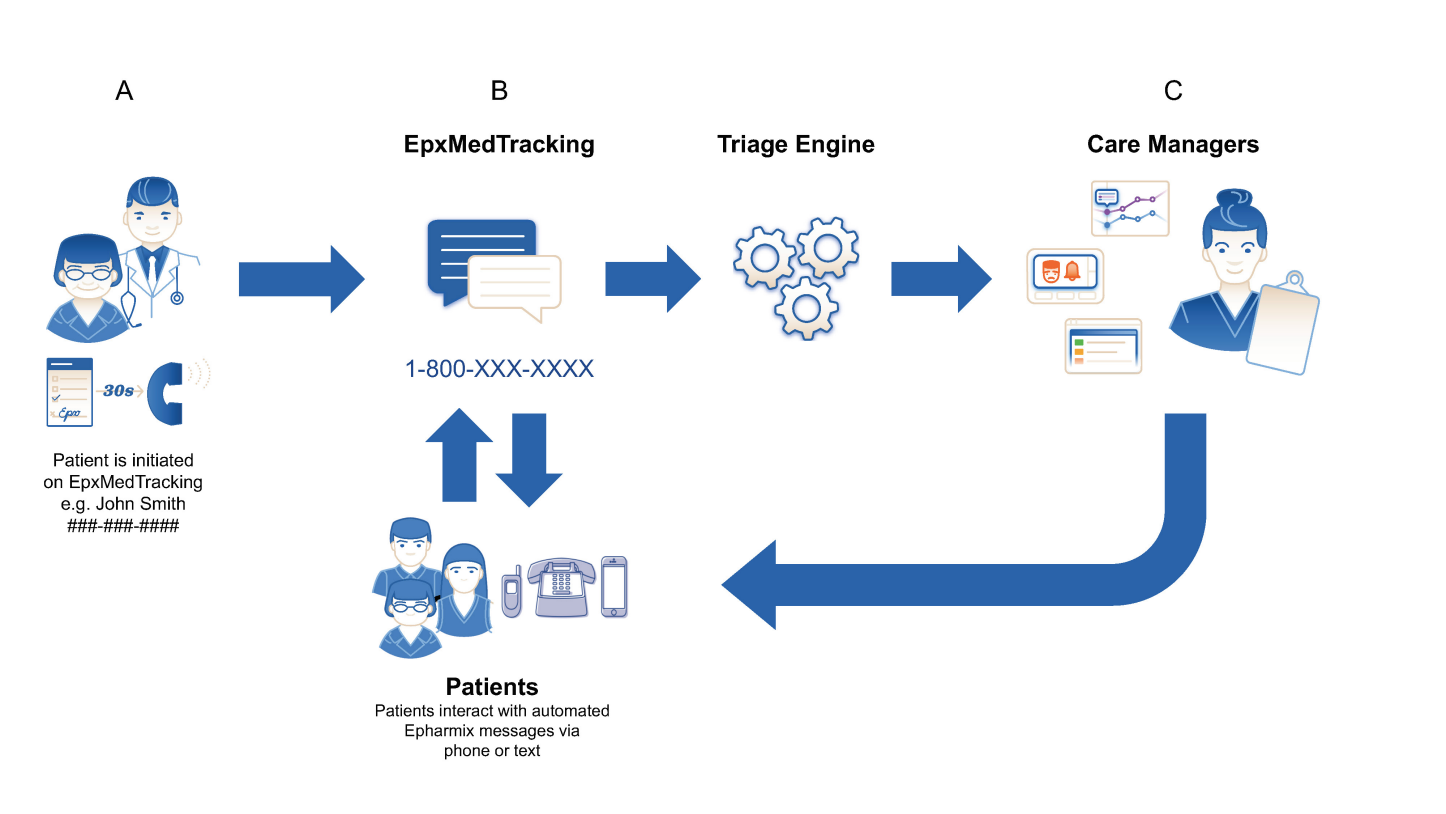
The workflow of messages and care managers used in EpxMedTracking. (A) Patients are initiated on EpxMedTracking with verbal consent to use. (B) EpxMedTracking assesses medication adherence and common issues. (C) A triage engine determines which patients can be aided in medication adherence (eg, running out of medication, a medication side effect) and care managers reach out to close the loop. Figure courtesy of Epharmix.

**Figure 3:**
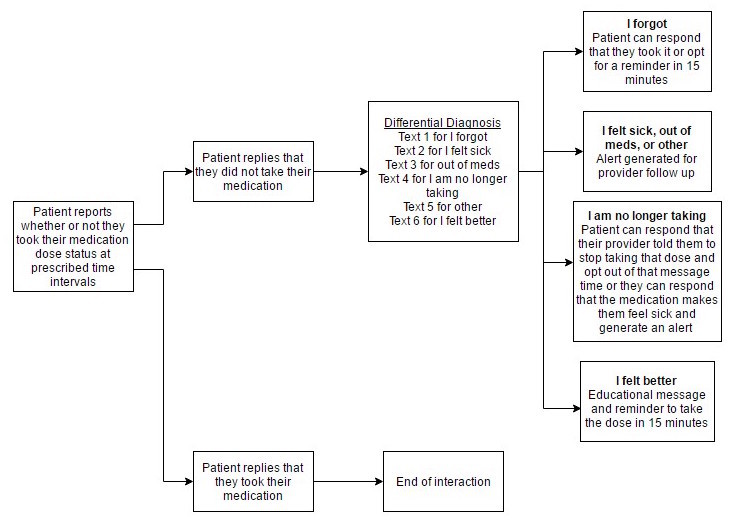
Text message workflow of the EpxMedTracking system.

EpxMedTracking implemented a medication adherence assessment of patients that then engaged providers using a triaged report and alert system developed by Epharmix. Functionally, EpxMedTracking assessed patient medication adherence via question and then triaged for common possibilities of nonadherence. Based on responses, it then reported back to the provider actionable information to facilitate provider intervention and rapid return to an appropriate medication regimen. Provider workflow of EpxMedTracking is depicted in Figure 2. A more detailed workflow of the text messages is depicted in Figure 3.

The frequency of messages was determined by the provider based on the frequency of medication doses that patients were required to take. For this study, care providers were free to access the Epharmix platform as often as they saw fit and were free to offer assistance to patients with issues in whatever way they deemed appropriate. No prompts were used to remind either patients or providers to use the app. Epharmix staff trained and supported providers using the software. Providers introduced the Epharmix software to their patients and trained them to use it as they saw fit.

### Outcomes

Our primary outcome was patient engagement rate which was calculated as the number of unique patients who responded at least once divided by the number of unique patients with sessions scheduled in a given time frame (weeks or days). Secondary outcomes included the reported reasons for missing doses in the pilot and the study period and self-reported medication adherence in the study period. Self-reported medication adherence was calculated as the number of patients reporting that they took their medication on a given day divided by the number of total sessions in that day. Patients were considered nonadherent if they reported missing a dose or did not respond to the message. Patients were able to respond to a message and report adherence to a given dose of medication at any point between when they received the medication reminder text for the dose in question to the time in which they received their medication reminder text for their next dose. Normalized days 74 to 76 were excluded from the graph because they included data from fewer than 10 patients. Message engagement, reasons for missing, and medication adherence data were collected by querying the Epharmix server.

### Sample Size

We did not calculate a necessary sample size for this study. We used convenience sampling of all of the patient data generated to date from the EpxMedTracking system.

### Statistical Methods

Patients who were deleted by their provider, opted out of messages on their own, or had a scheduled stop in the delivery of messages were included in the calculations of engagement rate, missed doses, and self-reported adherence until the point at which they stopped receiving messages. Patients who did not respond to the app but continued to receive messages were included in all calculations.

### Ethics and Informed Consent

Because this was a service improvement project and postmarket evaluation of an existing system and not an institutional review board–approved trial, there were no informed consent procedures for enrollment into the study. The data analyzed was de-identified to protect patient privacy. Best practices in data protection and Health Insurance Portability and Accountability Act compliance were used by Epharmix in the use of communications with patients.

## Results

**Figure 4:**
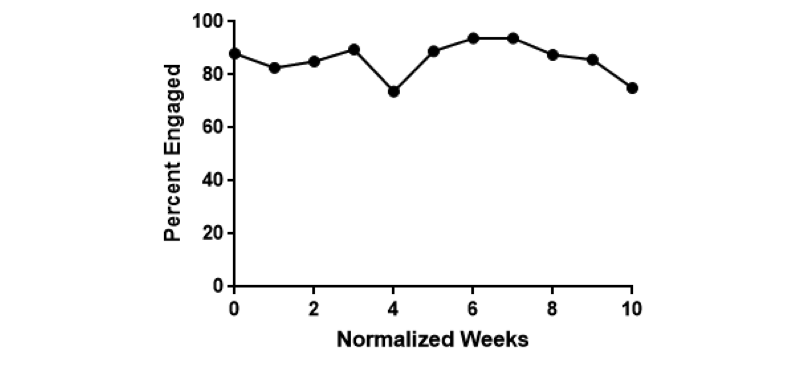
Patient engagement rates to EpxMedTracking normalized by week during the study period.

**Figure 5:**
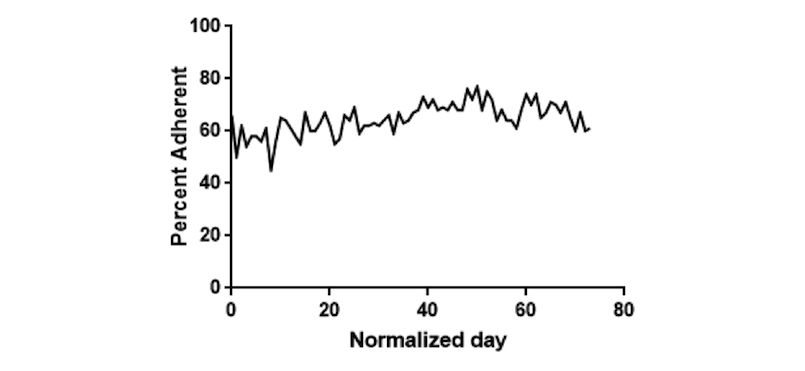
Patient engagement rate over time normalized by day.

A total of 2065 sessions were analyzed from 25 different patients over the study period. Of the 25 patients, 3 never responded to the system. Of those 3 unresponsive patients, 2 patients were deleted and 1 patient opted out during the study period. The engagement rate normalized to weeks on the system was calculated (Figure 4). There were 198 unique patient-week combinations over the 11 weeks. Of those 198 patient-weeks, a patient was enrolled but did not respond at all in a given week 28 times for an overall weekly engagement rate of 85.9%. The engagement rate over the same time period normalized by day is presented in Figure 5. This overall engagement rate was higher than the overall weekly engagement rate observed in the pilot, which was 73.3% (51 patients enrolled but not responding out of 191 patient-weeks).

**Figure 6:**
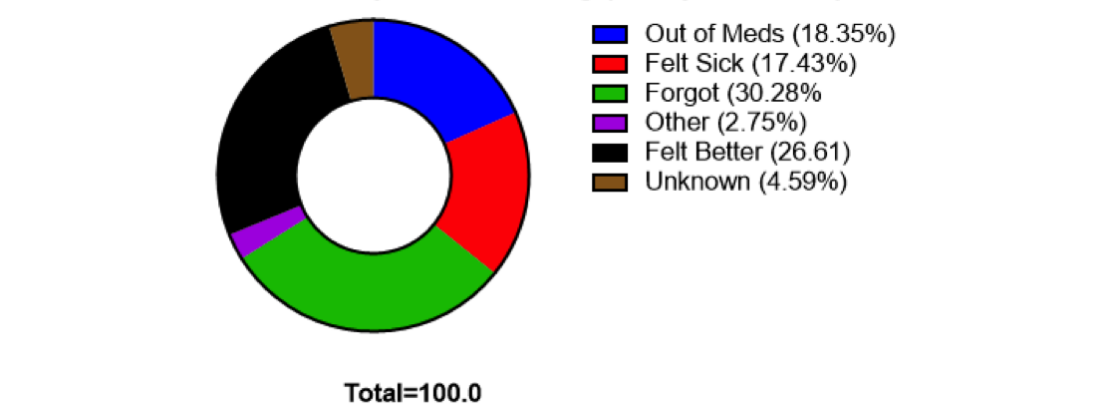
Reasons reported for missing medication doses in both the pilot and study period of EpxMedTracking.

**Figure 7:**
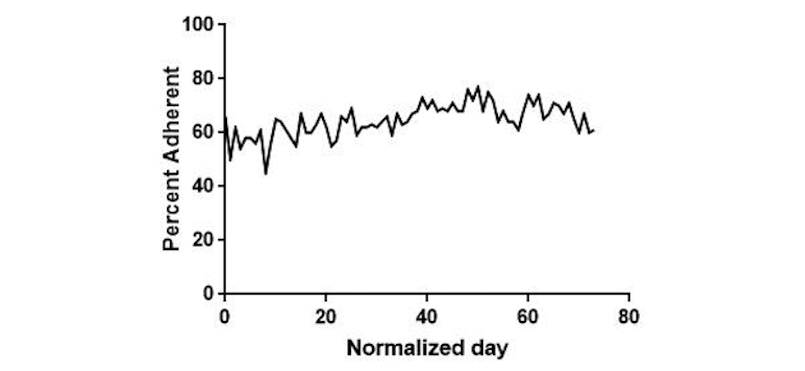
Self-reported medication adherence of patients who responded to the EpxMedTracking system.

There were 109 reported missed doses (Figure 6). The causes for missed doses in order of frequency were “I forgot” at 33 events (30.3%), followed by “I felt better” at 29 events (26.6%), “out of meds” at 20 events (18.4%), “I felt sick” at 19 events (17.4%), and “other” at 3 events (2.8%). No patients responded with “I am no longer taking.” An additional 5 missed doses occurred where the patient responded that the medication was not taken but they did not respond why. Such events are labeled in the chart as “Unknown.” We also noted an overall increase in self-reported medication adherence in patients using the EpxMedTracking system during the study period (Figure 7).

## Discussion

### Principal Findings

We set out to evaluate the feasibility of the EpxMedTracking system as a tool to improve medication adherence in terms of its acceptability by patients and its ability to determine why patients are missing doses. Our data suggest that the EpxMedTracking system is well received by patients as demonstrated by the high engagement rate of 85.9% (Figure 4) observed throughout the study period. This engagement rate remains high across the 11-week study period and never drops below 75%, indicating the EpxMedTracking intervention may be an effective tool for the longitudinal management of medication adherence in chronic conditions, which as mentioned previously are particularly susceptible to nonadherence [2].

A distinct strength of our system seems to be detecting when there is an actionable problem with a patient’s regimen, as 62.4% of the missed doses were due to “out of meds,” “felt better,” or “felt sick” (Figure 6). Importantly, this indicates that the EpxMedTracking system can be used to direct provider attention to where it is both needed and most useful, thus making the system well suited to improve the efficiency of provider time and clinical outcomes in a wide range of disease states where nonadherence may be an issue.

Improvements in self-reported adherence on the EpxMedTracking system indicate that enrollment in this system may also be able to change patient behavior over time (Figure 7). Future studies will look to expand on this possibility and see if system adoption corresponds to changes in relevant disease outcome measures such as viral titers in HIV patients.

### Comparison With Prior Work

In an analysis of phone call and SMS medication adherence interventions by Kashgary et al [6], many SMS- and phone call–based interventions to date have not been automated and required direct or manual messaging by a human provider [14,15]. This makes them useful for certain high-risk populations but prohibitively expensive and time-intensive in most clinical contexts. Of those that are automated, interventions often use only basic reminding [16] and thus only address the forgetfulness aspect of the complex and multidimensional problem of nonadherence, which only accounts for 30.3% of missed medication reasons in our sample. As such, the relatively low frequency of “I forgot” responses (30.3%) seems to support the theories of Saberi et al [10] and Brown et al [1] discussed above as they claim nonadherence is a complex, multidimensional problem that is not sufficiently explained by just forgetfulness.

While the technology and intervention described here are not novel, the nature of the intervention is novel. To date, the EpxMedTracking system is the first SMS-based intervention to both provide functional medication reminders and categorically identify problems leading to medication nonadherence, while simultaneously facilitating bidirectional patient-provider interaction. The categorical identification of patients who have actionable problems related to nonadherence allows the system to functionally identify and separate those nonadherent patients who would benefit from provider attention from those who are merely forgetful. In theory this should lead to more targeted and effective provider intervention in the realm of medication adherence and improved patient outcomes in a way that would be impossible with other text message–based medication adherence systems. As such, our subsequent work will evaluate the effectiveness of the EpxMedTracking system in terms of patient outcomes.

Given the improvement in overall engagement noted during our iterative development of EpxMedTracking from the pilot, this study shows that it might be possible that patient behavior can be affected by variations in wording and messages. This importantly could indicate that 2 digital health interventions with largely the same substance may produce significantly different results if they use slight variations in messages and wording. As such, it may be difficult to accurately make broad claims about eHealth and digital health. In order to establish evidence-based protocols for optimizing eHealth in the future, it may be wise to evaluate all digital health and eHealth interventions narrowly and in terms of the specific wording they use. This is also relevant in the context of prior work as it may provide an explanation for any inconsistencies existing in the current literature.

Unlike many other eHealth interventions, the EpxMedTracking system has the added strengths of being highly accessible for even the most socioeconomically disadvantaged patients because the system is inexpensive to operate and all messages are free to patients. The messages are accessible by anyone with a landline or cell phone, and no smartphone is necessary. This stands as a significant advantage of the EpxMedTracking system and other SMS- or phone call–based systems over mobile device app-based systems. App-based systems are often more complex and require smartphones and Internet connectivity which may cause problems with elderly or socioeconomically disadvantaged populations [17]. Additionally, apps may be less prone to patient engagement because a user must continue to choose to use the app, whereas with SMS or phone calls, patients must actively opt out of receiving messages or continuously ignore incoming messages [17].

### Generalizability

Because our analysis includes data from multiple commercial accounts, our data thus necessarily includes multiple different providers and styles of practicing medicine. This indicates that the results detected in this study are not provider- or practice setting–specific and externally valid for other organizations. Given that our data come from use in routine clinical practice, there are no specially structured elements specific to this study that could have an effect on use, adoption, or outcomes outside of this study setting.

### Limitations

Although only 30.3% of participants reported missing doses because they forgot, it is important to realize that there are alternative explanations for this finding based on the limitations of the study. For example, it is possible that this finding could at least partially be explained by patients not wanting to confess that they forgot to take their medications to their provider, instead perhaps opting to not respond or even deliberately choosing a false response. However, the fact that 30.3% of the time patients reported missing a dose because they forgot when there is no appreciable incentive for doing so suggests that patients engaging with EpxMedTracking probably respond as truthfully as they would in person.

It is also tempting to speculate that patients may feel less compelled to respond when they remember to take their medication and instead use the system for basic reminding and signaling to their provider when there is a problem with their regimen. Thus it is possible that the actual engagement rate could be higher than that observed in this study. In the future, we hope to explore these possibilities and better understand the patient experience using EpxMedTracking by administering anonymous surveys and comparing patient responses to medication refill data to validate the accuracy of the system and the honesty of patients using it.

While the results of this study show that enrollment in EpxMedTracking may be able to improve patient medication adherence over time (Figure 7), it is also possible that enrollment in telehealth interventions in general may be able to produce those kinds of changes. Because we obtained de-identified information, we also cannot comment on the patient demographics or disease states included in this study and the applicability of EpxMedTracking to different patient populations. Because patients come from both Missouri and Georgia, we do at least know that these findings are not community-specific. We also used convenience sampling for all of our analysis, and there was no control group and no washout period in between our pilot and the study period. Many of these limitations are inherent in a service improvement project with the currently available data, and we hope to address them in the future with more comprehensive trials.

### Conclusions

The evidence presented here shows that the EpxMedTracking system is a feasible tool that remains reliable over time and is useful for tracking self-reported medication adherence and identifying actionable problems with medication adherence in real time. Our data also raises the possibility that the wording and message algorithms of eHealth interventions might affect patient engagement and behavior, which would have important implications for the design of future eHealth interventions as well as the evaluation of the eHealth literature to date.

Despite a growing appreciation for the potential of eHealth in improving medication adherence, much work remains to be done before we realize its full potential. Further understanding in these areas has the ability to radically change our understanding of how patients and providers interact and shift the paradigm of how we monitor and treat medication nonadherence as well as countless other conditions.

## Appendix

Multimedia Appendix 1 Smart engagement module rotating greeting messages.

## Abbreviations

SES: socioeconomic status
SMS: short message service
