# Supplementary material for: EpxMedTracking: Feasibility Evaluation of an SMS-Based Medication Adherence Tracking System in Community Practice
Source: JMIR Res Protoc. 2017 May 15;6(5):e87. doi: 10.2196/resprot.7223 (PMC5447823; doi:10.2196/resprot.7223)
Supplement: Multimedia Appendix 1 [file resprot_v6i5e87_app1.pdf]

7223 supplementary table 1

Supplementary Table 1. Rotating greeting messages used in the smart engagement module
We're so excited to hear from you.
Thank you so much for answering our questions.
We're so excited to see your answer.
We'd love to hear from you.
We're always here for you.
We're so excited to see how you're doing.
We hope you're doing well.
We hope you're having a great day.
Let's keep working toward a happier and healthier you!
It's a great day to make decisions that can positively impact your health!
